# Interactions between the Kynurenine and the Endocannabinoid System with Special Emphasis on Migraine

**DOI:** 10.3390/ijms18081617

**Published:** 2017-07-30

**Authors:** Gábor Nagy-Grócz, Ferenc Zádor, Szabolcs Dvorácskó, Zsuzsanna Bohár, Sándor Benyhe, Csaba Tömböly, Árpád Párdutz, László Vécsei

**Affiliations:** 1 MTA-SZTE Neuroscience Research Group, University of Szeged, H-6725 Szeged, Hungary; gabor.balazs.nagy@gmail.com (G.N.-G.); zsuzsanna.bohar@gmail.com (Z.B.); 2 Faculty of Health Sciences and Social Studies, University of Szeged, H-6726 Szeged, Hungary; 3 Institute of Biochemistry, Biological Research Center, Hungarian Academy of Sciences, H-6726 Szeged, Hungary; zador.ferenc@gmail.com (F.Z.); dvoracsko.szabolcs@brc.mta.hu (S.D.); benyhe.sandor@brc.mta.hu (S.B.); tomboly.csaba@brc.mta.hu (C.T.); 4 Department of Neurology, Faculty of Medicine, Albert Szent-Györgyi Clinical Center, University of Szeged, H-6725 Szeged, Hungary; apardutz@yahoo.com

**Keywords:** cannabinoids, endocannabinoids, cannabinoid receptors, kynurenines, opioids, migraine

## Abstract

Both the kynurenine and the endocannabinoid systems are involved in several neurological disorders, such as migraine and there are increasing number of reports demonstrating that there are interactions of two systems. Although their cooperation has not yet been implicated in migraine, there are reports suggesting this possibility. Additionally, the individual role of the endocannabinoid and kynurenine system in migraine is reviewed here first, focusing on endocannabinoids, kynurenine metabolites, in particular kynurenic acid. Finally, the function of NMDA and cannabinoid receptors in the trigeminal system—which has a crucial role in the pathomechanisms of migraine—will also be discussed. The interaction of the endocannabinoid and kynurenine system has been demonstrated to be therapeutically relevant in a number of pathological conditions, such as cannabis addiction, psychosis, schizophrenia and epilepsy. Accordingly, the cross-talk of these two systems may imply potential mechanisms related to migraine, and may offer new approaches to manage the treatment of this neurological disorder.

## 1. Introduction

The endocannabinoid system is involved in several neurological pathological conditions including neuropathic pain, inflammatory diseases, movement disorders (Parkinson’s disease and Huntington’s disease) and multiple sclerosis [1,2,3]. Cannabis has been used for a long time to treat nausea and vomiting, and to treat pain and migraine since the 6th century [4]. Migraine is one of the most prevalent neurological disorders, which affects about 16% of the population [5]. The total cost of healthcare for patients with migraine in Europe in 2010 was 18.4 billion € [6]. Growing evidence implies that endocannabinoid and glutamatergic systems are connected to migraine pathophysiology.

Human and animal data show that migraine is presumably hyperexcitability disorder, which means that the glutamatergic system is overactive [7]. In addition, an increasing amount of evidence suggests that migraine could alsobe linked to the kynurenine pathway (KP) itself [8].

Endocannabinoids, also known as “the body own cannabinoids” [9], and its receptors, have an extensive link with other endogenous receptors, such as opioid and glutamate ones, and especially the *N*-methyl-d-aspartate (NMDA) receptors. One of the endogenous NMDA receptor antagonists is kynurenic acid (KYNA), which is generated through tryptophan (Trp) metabolism. KYNA has a neuroprotective function and it might prove to be a future candidate in the treatment of migraine possibly by its NMDA antagonism.

The aim of this review is to demonstrate the interaction between the endocannabinoid and kynurenine system in relation to migraine. The review will discuss the involvement of each system in migraine separately, focusing on glutamate, kynurenines, endocannabinoids and the role of NMDA and cannabinoid receptors in the trigeminal system. Finally, we will cover the already demonstrated or possible interactions of the kynurenine and endocannabinoid systems, which can be potentially relevant to migraine.

## 2. Glutamate and Migraine

Glutamate is an ionic form of the nonessential amino acid glutamic acid, and it is the main excitatory neurotransmitter in the central nervous system [10]. Thus, it excites nearly every neuron contributing the primary neural transmission and pain perception [11,12]. As a neurotransmitter, glutamate is synthesized from glutamine, by the mitochondrial enzyme glutaminase, and is stored in synaptic vesicles. During neurotransmission, it is released from the stores to the synaptic cleft and removed by the presynaptic glutamate transporter and the transporter located on the neighboring glial cells. In glial cells, glutamate is converted to glutamine by glutamine synthetase. Thereafter, glutamine is transported out of the glia and picked up by nerve cells and transformed to glutamate [10].

Glutamate receptors can be divided into ionotropic and metabotropic receptors. The ionotropic receptors, namely NMDA, α-amino-3-hydroxy-5-methyl-4-isoxazole propionic acid (AMPA) and kainate receptors are ligand-gated ion channels. The metabotropic receptors are G-protein coupled receptors (GPCRs), which mean that their activations depend on a biochemical cascade [13].

Glutamate excitotoxicity is related to the hyperexcitability of NMDA receptors, as described in 1969 by Olney [14]. During this process, high glutamate stimulation leads to a large amount of Ca^2+^ is entering the cell [15] influencing many enzyme functions, such as phospholipases, proteases and endonucleases [16]. These mechanisms have a pivotal role damaging cell structures and DNA causing neuronal cell death. These receptors, especially the NMDA receptors have a crucial role in the pathomechanisms of migraine [17], supported by various experimental observations showing increased levels of glutamate in plasma, cerebrospinal fluid and platelets in migraineurs [18,19,20].

Glutamate has also a relevant role in the peripheral and central sensitization of the trigeminal system, is crucial in the pathomechanism of migraine [21]. Activation of NMDA receptors is one of the most important steps in initiating and maintaining the central sensitization [22], which can be blocked by competitive (D-CPP) and non-competitive (MK801) NMDA receptor antagonists in rats [23]. In addition, a conditional deletion of the NR2 subunit of NDMA receptors inhibits the synaptic inputs through NMDA receptors and the central sensitization in rats [24]. Besides NMDA, the metabotropic glutamate receptors also contribute to the mechanical allodynia [25]. Data from the human studies showed that the levels of glutamate were higher in the plasma, cerebrospinal fluid and platelets in migraine patients compared with non-migraineurs [20,26,27], which could indicate an increased activation of glutamate receptors, thus hyperexcitability [8].

## 3. Kynurenine Pathway (KP) and Migraine

**Figure 1:**
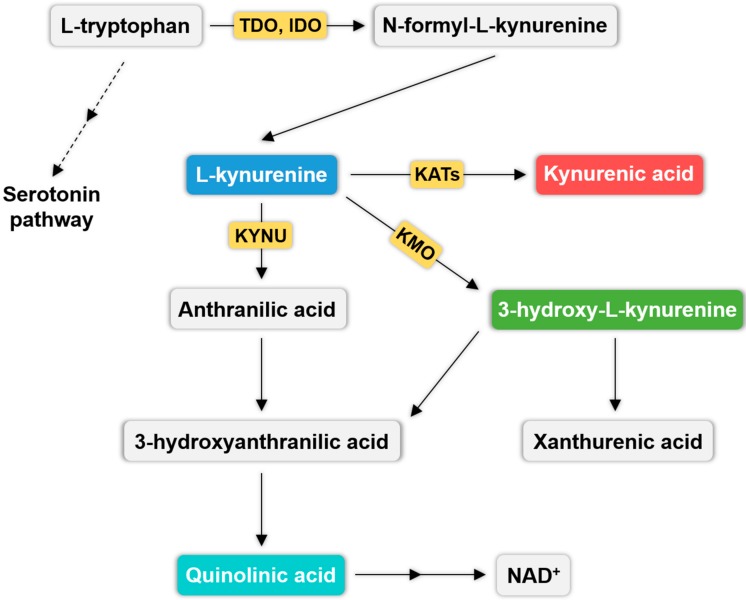
The kynurenine pathway. The most relevant metabolites of the pathway are highlighted in different colors, and the key enzymes are also represented on the arrows. Dashed arrows indicate the indirect linkage between the kynurenine and serotonin pathway. The two sequentially arrows symbolizes the multiple (not indicated) steps in quinolinic acid metabolization to nicotinamide adenine dinucleotide (NAD^+^). Abbreviations: IDO (indoleamine 2,3-dioxygenase); KATs (kynurenine aminotransferases); KMO (l-kynurenine 3-monooxygenase); KYNU (l-kynurenine hydrolase); NAD^+^ (nicotinamide adenine dinucleotid), TDO (tryptophan 2,3-dioxygenase).

KP is a dominant part of Trp metabolism, since 95% of Trp metabolizes this way. Trp, an essential amino acid, is transformed to *N*-formyl-l-kynurenine by tryptophan 2,3-dioxygenase (TDO) and indoleamine 2,3-dioxygenase (IDO), which are the rate-limiting enzymes of the KP. *N*-formyl-l-kynurenine can be further metabolized by formamidase to l-kynurenine (l-KYN), which is the precursor of KYNA (synthetized by kynurenine aminotransferases (KAT)s). l-KYN can also be degraded to anthranilic acid (ANA) by l-kynurenine hydrolase (KYNU) or to 3-hydroxy-l-kynurenine (3-HK) by kynurenine 3-monooxygenase (KMO). ANA and 3-HK are then further transformed to 3-hydroxyanthranilic acid (3-HA), which metabolizes to quinolinic acid (QUIN). 3-HK can be converted to xanthurenic acid as well. In thelast step of KP, QUIN is converted to nicotinamide adenine dinucleotide (NAD^+^) (Figure 1).

Among the KP metabolites, many compounds are biologically active. 3-HK and 3-HA are able to raise the formation of free radicals, yielding oxidative stress [28]. KYNA can exert its effect through NMDA and other glutamate receptors, namely AMPA [29] and kainate receptors [30].

In addition, KYNA has an agonistic effect of on the G protein coupled receptor 35 (GPR35) [31], which was found for a long time only in the gastrointestinal and in the immune system, namely in the crypts of Lieberkühn [32]. However, recent experiments showed that this receptor can also be found in the nervous system [33], and it has a relevant role in pain processing and neuroinflammation [34].

Opposite to KYNA, QUIN is an agonist of NDMA receptors and it can induce neuronal cell death [35] and lipid peroxidation [36]. In addition, QUIN is able to inhibit glutamate uptake in rats, resulting in raised extracellular glutamate levels [37] (Figure 1).

Several animal studies indicate that kynurenines, its analogs and halogenated derivatives have a future potential therapeutic action in the treatment of migraine. Since KYNA has a poor ability to cross the blood-brain barrier, its analogs and derivatives are tested experimentally. Halogenated derivatives, 4,6-dichlorokynurenine and 4-chlorokynurenine are converted to KYNA derivatives (7-chlorokynurenic acid and 5,7-dichlorokynurenic acid), which have increased affinity to the glycine-binding site of NMDA receptors [38,39].

One of the human and animal models of migraine is the administration of nitroglycerin (NTG), which is a nitric oxide (NO) donor. NTG is able to activate and sensitize the trigeminal system, which are the crucial mechanisms in the pathophysiology of migraine [40,41]. Administration of l-KYN and probenecid (an inhibitor of KYNA secretion from the central nervous system (CNS) together or KYNA analogs ((*N*-(2-*N*,*N*-dimethylaminoethyl)-4-oxo-1*H*-quinoline-2-carboxamide hydrochloride (KA1) and *N*-(2-*N*-pyrrolidinylethyl)-4-oxo-1*H*-quinoline-2-carboxamide hydrochloride (KA2)) were effective to inhibit the NTG caused morphological and behavioral changes in rats likely via the inhibition of NDMA receptors [42,43,44]. In this model, NTG was able to decrease the expression of kynurenine aminotransferase II (KATII) [45], which is the key enzyme of KYNA production. In a recent study, it was also shown that NTG altered the expression of other enzymes of the KP, namely TDO, IDO, KYNU and KMO, suggesting that NTG/NO has an influence on the KP [46].

Another animal model of trigeminal activation and sensitization is the application of Complete Freund’s Adjuvant (CFA) on the dural surface causing inflammation. In this experimental setting, Lukács and her colleagues showed that KA1 was able to attenuate the CFA-caused inflammation [47].

The orofacial formalin test is also a suitable model for mimicking the trigeminal activation and sensitization [48]. In this model, probenecid decreased the nociceptive behavior in rats, probably via increasing the concentration of KYNA [42]. In a recent study KA1 and KA2 were able to abolish the formalin induced behavioral and morphological changes, and increased KYNA levels [49]. On the other hand, in the combined model of NTG and formalin, KA1 also inhibited the behavioral and morphological alterations [50]. In addition, in the electrical stimulation model of the trigeminal activation a decreased KAT immunoreactivity has been shown in the rat’s dura mater [51].

Cortical spreading depression (CSD) is a self propagating wave in the cortical areas of the brain and it has a relevant role in the pathomechanism of migraine [52], as it is well accepted that CSD is the basis of the aura phenomena [53]. In the model of CSD, two new KYNA analogs inhibited the propagation of CSD waves [54], likely by the inhibition of glutamate receptors. Since glutamate receptors have a crucial role in the propagation and generation of CSD [55], they possibly represent a link between migraine and CSD.

It is also important to note that, the levels of the metabolites of the KP were found to be changed in migraineurs. Curto and her colleagues discovered decreased level of kynurenine metabolites in the serum of patients with chronic migraine and cluster headache [56,57], the data of which are in accordance with animal results from the NTG model of migraine [45]. These findings suggest that the decreased levels of KYNA mean that the glutamatergic system is overactive in chronic migraine as well as cluster headache.

The role of KYNA and its metabolites in the pathomechanisms of migraine is still not fully known. The effect of KYNA could manifest through both peripheral and central mechanisms. On the periphery, KYNA can modulate glutamate receptors, principally, NMDA receptors localized in the dorsal root and trigeminal ganglia [58]. The other peripheral place, where KYNA and its analogs can exert their effect is GPR35, which is also present in the dorsal root ganglion (DRG) [59]. Besides the peripheral effects, KYNA and its analogs have an impact on second-order neurons too, proven by that KYNA can decrease mechanical allodynia and pain sensitivity in the hot-plate and tail-flick tests [60,61].

To summarize the human and animal data, we can conclude that the KP has a relevant role in the pathomechanism of migraine, and it might be promising future therapeutic target in the treatment of headaches.

## 4. The Endocannabinoid System and Migraine

### 4.1. The Endocannabinoid System

The endocannabinoid system comprises of the endogenous ligands called endocannabinoids, the enzymes which synthesize and degrade them and the receptors, to which these ligands bind. Two types of cannabinoid receptors have been cloned so far, the type 1 and type 2 cannabinoid receptor (CB1 and CB2) from rat cerebral cortex [62] and human promyelocytic leukaemia cells, respectively [63]. They are class A GPCRs and belong to the G_i/o_-coupled GPCR superfamily and they couple to G_i/o_ type inhibitory G-protein, thus their activation inhibits cyclic adenosine monophosphate (cAMP) production and stimulates mitogen-activated protein kinases (MAP). The CB1 receptor is the most abundant GPCR in the CNS and its density is comparable to that of the glutamate, γ-Aminobutyric acid (GABA) and dopamine receptors [62,64,65]. It can also be found in the periphery such as in the liver, adipose tissues, muscles, cardiovascular and gastrointestinal system [66,67]. The CB2 receptors are mainly present on immune and hematopoietic cells [2,68], but can also be found in the CNS especially in microglia [69], in the periphery on myocardial cells [2,66] and in the endothelium [66,70]. CB1 receptors are responsible for mood regulation and can also induce antinociception, regulate energy balance and endocrine functions [66,67,71]. The major function of the CB2 receptor is the control of cytokine release and immune cell migration (reduce inflammation-induced pain, reveal peripheral antinociception, inhibition of tumor growth) [72]. CB2 receptor can decrease nociception so far without any detectable tolerance [72,73] and side effects [74]. Activation of this receptor caused analgesia in the tail flick and the orofacial formalin test [75], as well.

Endocannabinoids are lipid-derived hydrophobic compounds, among them *N*-arachidonoylethanolamine (anandamide, AEA) and 2-arachidonoylglycerol (2-AG) are the most studied [76,77,78]. AEA is a full agonist of CB1 and partial agonist of CB2 receptors. AEA is synthesized from membrane phospholipids via *N*-acyl transacylase and *N*-acyl phosphatidylethanolamine-phospholipase D [79]. In the degradation of AEA is proceeded mainly by fatty acid amide hydrolase (FAAH), which degrades AEA to arachidonic acid and ethanolamine [79,80]. Blocking the FAAH enzyme by irreversible inhibitors such as URB597 has been reported to be a promising treatment for smoking addiction [66,81,82] and it also enhanced opioid analgesia [83,84]. 2-AG is formed from the omega-6 fatty acid arachidonic acid and glycerol [79]. 2-AG can be found in relatively high concentration in the nervous system [85] and it is mainly degraded by monoacylglycerol lipase (MAGL) [86,87].

### 4.2. The Role of Endocannabinoids in Migraine

Cannabis has been used for migraine medication since 6th century and the deficiency of endocannabinoid system contributes to the pathophysiology of the disorder [4]. This system has a crucial role in the pathomechanisms of pain [88] and its activation is essential in the inhibition of trigeminal neurons [89].

Clinical studies shown that the formation of AEA and 2-AG were down-regulated in migraineurs [90,91]. In patients with chronic migraine a decreased AEA level was found in the cerebrospinal fluid [92], as well. In addition, in the blood of female migraine patients, a raised FAAH enzyme level and a decreased AEA level has been shown [93]. The reduced levels of AEA might promote the hyperactivity of the trigeminal system and the reduced inhibitory impact of the endocannabinoid system [94,95], both contributing to migraine development.

Numerous studies show that endocannabinoids are effective in the animal models of migraine. Endocannabinoids control the cerebrovascular tone and contribute to NO production [88]. In the NTG model of migraine, AEA was able to inhibit the NTG induced increase of c-Fos expression in rats [95], one of the markers of trigeminal neuronal activation. Besides that, AEA can also abolish the NTG caused elevation of the levels of the sensitization markers in rats [45] and also inhibit the NO induced dural vasodilatation [96]. AEA is effective in the inhibition of the NTG induced KAT-II expression decrease [45], suggesting an influence on the KP. In these above-mentioned experiments, the main modulatory effect was probably achieved by the CB1 receptor, which can be activated by increased AEA levels [97]. This notion is supported by the experiments in which CB1 receptor activation was able to alleviate the KCl-induced CSD [98] and repressed the Aδ neuron activity in rats [99]. In addition, AEA can alter NO and calcitonin gene-related peptide (CGRP) induced dural vasodilatation [96]. CGRP is the main peptide in the pathomechanisms of migraine, its level is raised in external jugular veins in migraineurs [100] and it co-localizes with CB1 receptors [88]. On the other hand, 2-AG created an anti-nociceptive effect in the formalin test [101] and endocannabinoid uptake inhibitors were effective in pain relief in the formalin test [102], which shows that the levels of endocannabinoid are important in the pain inhibition process. WIN55121, a potent cannabinoid agonist can inhibit the wind-up process, which is a centrally mediated enhance of C-fibers and contributes to the improvement of allodynia [103].

The molecular or genetic inhibition of the AEA degradation enzymes enhance cannabinoid signaling and raise the levels of AEA in the brain [80], thus making an opportunity to influence the levels of the endocannabinoids in many experimental protocols. Indeed, FAAH inhibitors have an analgesic effect in the inflammatory and the neuropathic models of pain [104]. In addition, NTG was able to enhance the enzyme levels of FAAH and MAGL [95], thus contributed to the decreased AEA and 2-AG levels in animals.

## 5. Cannabinoid and Glutamate Receptors in the Trigeminal System

In migraine pathomechanism, trigeminal system has a pivotal role. The trigeminal system consists of the peripheral Aδ and C-fibers, which convey to the trigeminal ganglion from the peripheral skin and meningeal blood vessels. The brainstem includes the second-order trigeminal neurones, which receive nociceptive afferents from trigeminal ganglion and modulating afferents from other brainstem structures, as locus coeruleus, nucleus raphe magnus and periaqueductal grey (PAG), which also called migraine generators. The information from brainstem nuclei proceeds to the somatosensory cortex via the third-order neurons located in the thalamus.

Cannabinoid receptors can be found throughout the trigeminal system and in the migraine generators, as well. In the trigeminal system CB1 is present in the trigeminal ganglion, in the rostral and caudal PAG and on the peripheral and central axon terminals of trigeminal primary sensory neurons [88]. They are also present in the human or rat thalamus and PAG [105,106] and rostral ventromedial medulla [88], from where trigeminal system receives inputs. CB2 is also present in the nervous system, can be found in the afferent fibers in the dorsal horn of the spinal cord [107]. Thus, activation of these receptors may modulate the neuronal firing of the trigeminal system [94]. Activation of glutamatergic projections is able to facilitate the synthesis of endocannabinoids in the glutamatergic terminals. CB1 receptors are located on these terminals and their activation can decrease the excitatory transmission and glutamate induced hyperexcitability [108]. On the other hand, a genetic association study found strong haplotypic associations between the CB1 gene and the three prognostic symptoms of migraine, as disability, nausea and photophobia [109], suggesting that the haplotype causes diminished CB1 function or expression.

Glutamate receptors are also found in the trigeminal system. NMDA, AMPA, and kainate receptors are present within the brainstem nuclei of the trigeminal system [110] and NMDA receptor mRNA was found in the trigeminal ganglion [111]. NMDA, AMPA, and kainate receptors can also be found in the superficial layers of the spinal cord [112], where the brainstem trigeminal nuclei extend.

Several experimental data show that the levels of glutamate and endocannabinoid are altered in migraine patients. To summarize human and animal data with kynurenines and cannabinoids, we can conclude that they have a relevant role in the pathomechanisms of migraine, probably also by their interaction.

## 6. Known and Potential Functional Interactions betweenthe Endocannabinoid and Kynurenine System: Possible Pharmacological Targets against Migraine

Thus far there are rather limited data demonstrating the interaction between the kynurenine and endocannabinoid systems [108] and their implication in migraine has not yet been investigated at all. Nevertheless, the functional co-operation between the two systems may reveal other, yet unknown mechanisms, which might be involved in migraine and may display novel potential therapeutic targets. There are studies describing connections between exogenous cannabinoids and the enzymes of the KP, whereas other data demonstrate the anatomical and functional interactions between CB1 and NMDA receptor, in which KYNA is used more as a tool to indicate the involvement of the NMDA receptor in the described effects [108]. Both possibilities will be discussed in this section, together with potential interplay between the cannabinoid and further target receptors of KYNA. An additional interaction will also be proposed among the µ opioid, the CB1 and the NMDA receptor.

### 6.1. The Relationship between Enzymes of the KP and Exogenous Cannabinoids

Enzymes of the endocannabinoid and kynurenine system have been demonstrated to be potential therapeutic targets in several pathological conditions [113,114,115,116], including migraine [116,117]. Thus far no direct interaction has been described between these enzymes, however there issome evidence for overlapping expression profile in certain neurons. For instance, IDO and FAAH enzymes are both expressed in neurons of the hippocampus and dentate gyrus [118,119]. On the other hand, Jenny M. Santer et al. demonstrated that Δ9-tetrahydrocannabinol (THC) or cannabidiol (phytocannabinoids of the cannabis sativa plant) in nanomolar concentrations can enhance mitogen-stimulated IDO enzyme activity (Figure 1), which was dependent from CB1 or CB2 receptor activity. In contrast, in micromolar concentrations both compounds suppressed the activity of the IDO enzyme independently from cannabinoid receptors, consequently enhancing TRP levels for serotonin synthesis (Figure 1), which overall may contribute to improve mood disturbance [120]. Incidentally, cross-talks between the CB1 and serotonin (5-hydroxytryptamine) receptors has been also described previously [99,121,122].

Furthermore, Justinova and co-workers reported that by enhancing brain KYNA levels with a KMO (Figure 1) inhibitor (Ro 61-8048) attenuated cannabinoid-induced increase in dopamine levels in the nucleus accumbens (NAc) shell and thus reduced the rewarding effects of THC and WIN55,212-2 [123]. This study also demonstrated that the anti-abuse action of elevated brain KYNA levels by KMO inhibition is due to KYNA-induced negative allosteric modulation on the α7 nicotinic acetylcholine receptor (α7nAChR), since this effect was prevented by a α7nAChR selective positive allosteric modulator (PNU120596) [123]. Thus, the selective elevation of brain KYNA levels can be suggested a potential novel strategy for treating human marijuana dependence and it can be considered a pharmacologically safe approach since Ro 61-8048 has not been associated with adverse side effects [123].

Accordingly, cannabinoids can alter the activity of certain enzymes of the KP and vice versa, manipulating KYNA levels through enzyme inhibition in the kynurenine pathway can also alter exogenous cannabinoid activity. However, it is yet to be examined whether altering the levels of endocannabinoids for instance via FAAH or MAGL inhibitors induces any changes in the kynurenine pathway and vice versa.

### 6.2. The Type 1 Cannabinoid Receptor-N-methyl-d-aspartate (CB1-NMDA) Receptor Complex

Among the known receptor targets for KYNA, the NMDA receptor is the most cited and studied and therapeutically one of the most relevant targets in terms of neuropathic pain and migraine [116]. The NMDA receptor is a ligand-gated cation channel, permeable for monovalent and Ca^2+^ ions. The ion permeability is co-activated by glutamate and glycine through separate binding sites. NMDA receptors consist of three types of subunits, NMDA receptor subunit 1 (NR1), NMDA receptor subunit 2 (NR2) and NMDA receptor subunit 3 (NR3), which form a functional tetramer of an obligatory pair of NR1 and two NR2 or NR3 subunits [124]. KYNA binds to the glycine B binding site of the NMDA receptor [125] with micromolar affinity [126,127], antagonizing the effects of the receptor [128].

The CB1 and the NMDA receptor systems are involved in multiple processes, such as learning and memory [129,130,131], drug reinforcement [132,133,134], or nociception [88,135,136]. Nevertheless, the most widely studied and known pathological disorders in which these two receptors interact are psychosis, schizophrenia and epilepsy [124,137].

NMDA and CB1 receptors have been demonstrated to be post and pre-synaptically co-localized [138,139,140,141,142] allowing them to functionally interact in both sides of the synapse [143]. Moreover, a physical association has been previously described between the C-terminal of the CB1 receptor and NR1 subunit of the NMDA receptor [144]. Incidentally, NMDA receptors are known to interact physically with other GPCRs as well, such as the μ opioid receptor (MOR), dopamine D1 receptor, group 1 metabotropic glutamate receptor [145,146,147]. The CB1-NMDA receptor complex requires the presence of the histidine triad nucleotide-binding protein 1-σ receptor type 1 (HINT1-σ1R) protein tandem [142,148], which works as an on-off switch, connecting and disconnecting the two receptors, which can be regulated by calcium and exogenous σ1R ligands [149]. The HINT1-σ1R protein tandem has been described in the MOR-NMDA receptor complex as well [150].

It is now well-established that both endogenous and exogenous cannabinoids reduce the activity of NMDA receptors through the CB1 receptor [142,151,152] by reducing pre-synaptic glutamate release or alter post-synaptic NMDA receptor mediated signaling pathways [143,152] and it also requires both HINT1 and σ1R proteins [150,153]. Additionally, exogenous cannabinoids effectively induce CB1 receptor internalization, which disassembles and deactivates the CB1-NMDA receptor complex, dampening NMDA receptor activity, thus reducing the risk of NMDA receptor mediated excitotoxicity [142,154]. NMDA receptor hyperactivity is one of the main characteristics of epilepsy and NMDA receptor antagonists display antiepileptic effects in clinical and preclinical studies, unfortunately associated with serious side-effects, such as memory dysfunctions or motor disturbances [155]. Phytocannabinoids, such as THC and cannabidiol have been reported to reduce epileptic seizures, utilizing the CB1-NMDA receptor complex, thus introducing a possible new therapeutic approach for epilepsy [124]. However, excessively reducing NMDA receptor activity can lead to NMDA receptor hypofunction, which is implicated with psychosis and schizophrenia [137]. Indeed, cannabis abuse has been associated with these two mental disorders [156,157]. The HINT1 and σ1R genes have also been implicated in schizophrenia [158,159,160], moreover the σ1R agonist pregnenolone and antagonists showed promising results in reducing the symptoms of schizophrenia in clinical trials [161,162]. Additionally, the CB1-NMDA receptor complex has been implicated in analgesia as well. Although cannabinoid mediated analgesia does not require the association of CB1 receptor with the NMDA receptor, when coupled, NMDA receptor antagonists can significantly reduce cannabinoid-induced analgesia, the effect of which is HINT1 protein dependent [142].

**Figure 2:**
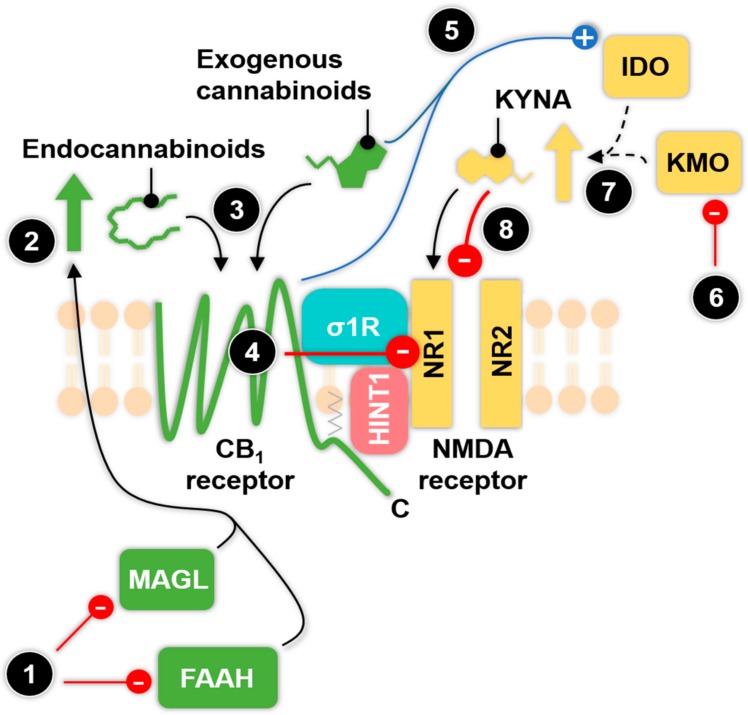
The type 1 cannabinoid receptor-*N*-methyl-d-aspartate (CB1-NMDA) receptor complex and potential pharmacological targets to reduce *N*-methyl-d-aspartate (NMDA) receptor hyperactivity, which is one of the main pathomechanism of migraine. The pharmacological inhibition (red arrow) of endocannabinoid metabolizing enzymes, monoacylglycerol lipase (MAGL) or fatty acid amide hydrolase (FAAH) (1) increases (upward green arrow) endocannabinoid levels (e.g., 2-arachidonoylglycerol (2-AG), anandamide (AEA)) (2) [113], thus enhancing the agonist-mediated type 1 cannabinoid receptor (CB1) receptor activity (3) (in this step the black arrows indicate ligand binding). This mechanism will overall reduce (red arrow) the activity of the NMDA receptor, hence the risk of excitotoxicity via CB1 receptor and the σ1R-HINT1 protein tandem (4) [124,153,154]. Exogenous ligands can also induce CB1-receptor mediated NMDA receptor inhibition more effectively (3,4) [124]. Additionally, exogenous cannabinoids such as cannabidiol or Δ9-tetrahydrocannabinol are known to stimulate (blue arrow) the indoleamine 2,3-dioxygenase (IDO) enzyme activity in dependence of cannabinoid receptor activation (5) [120]. This stimulation, together with the pharmacological inhibition (red arrow) of the kynurenine 3-monooxygenase (KMO) enzyme (6) [123] may enhance (yellow upward arrow) endogenous KYNA levels indirectly (indicated by dashed lines), through the kynurenine pathway (7) (Figure 1), which will result an enhanced reduction in NMDA receptor activity via the antagonizing effect (black arrow) of kynurenic acid (KYNA) (8).The figure shows a simplified, hypothetical scenario of the indicate elements and mechanisms of the endocannabinoid and kynurenine system within the CB1-NMDA receptor complex, which has been individually reported previously in other circumstances (cited accordingly). The figure also indicates the sigma 1 receptors- histidine triad nucleotide-binding protein 1 (σ1R-HINT1) protein tandem, which associates the two receptors and it is based on Rodriguez-Munoz and co-worker’s review [124]. NR1 and NR2 indicate the two types of subunit of the NMDA receptor and the C terminus of the CB1 receptor is also highlighted. The green color indicates endocannabinoid, while the yellow color indicates kynurenine system related ligands, receptors, enzymes or mechanisms. The shapes of the indicated ligands, receptors or enzymes are schematic or overly simplified representations of their structures.

Thus, the CB1-NMDA receptor complex, including the HINT1-σ1R protein tandem offers a promising new approach for the therapeutic management of certain neurological disorders [124]. NMDA receptor hyperactivity is one of the main pathomechanisms of migraine [8] and since CB1 receptor has been demonstrated to hinder this activity, the CB1-NMDA receptor complex could be a potential therapeutic target for migraine. Furthermore, based on the previous section and the data above mentioned, this can be extended by the inhibition of endocannabinoid metabolizing enzymes or by manipulating the enzymes of the kynurenine pathway. This way, the enhanced levels of endocannabinoids, or exogenously administered cannabinoids via CB1 receptor and/or enhanced levels of KYNA may represent an alternative approach for the reduction of NMDA receptor hyperactivity and thus against migraine (Figure 2). Additionally, the NMDA receptor being one of the key receptor target for KYNA, bifunctional KYNA-cannabinoid/σ1R ligands or co-administration of KYNA and cannabinoids might be a further therapeutic tool to utilize the CB1-NMDA receptor association in migraine or other neurological disorders.

### 6.3. Possible Interaction between the μ Opioid, the CB1 and the NMDA Receptor

There are extensive data describing the interaction between the kynurenine and the endogenous opioid system [60,163,164,165,166,167]. It has also been demonstrated that KYNA and its analog KA1 can indirectly alter the G-protein signaling of opioid receptors through the NMDA receptor depending on the opioid receptor type (μ, κ or δ) and brain region (cortex or striatum) [168,169]. More importantly, the opioid, cannabinoid and the NMDA receptors are known to be co-localized and functionally interact with each other pair-wise in areas relevant to opioid dependence, tolerance and antinociception [84,142,170,171,172,173,174,175]. Additionally, similar to CB1 receptor, MOR also physically associates with the NR1 subunit of the NMDA receptor, and it is also under the control of the HINT1 and σ1R proteins [149]. Interestingly, in contrast to CB1, MOR promotes the activity of the NMDA receptor in this complex [150]. Thus, KYNA might alter the functionality of cannabinoid and opioid receptors via the NMDA receptor and might form a functional “triangle”. Such a proposal has been reviewed previously between opioid, cannabinoid receptors and the transient receptor potential vanilloid type 1 (TRPV1) channel in terms of analgesia [176].

### 6.4. The Interaction between the CB1 and the α7nACh Receptor

There are numerous publications reviewing the interaction between the cholinergic and endocannabinoid system which has been reviewed elsewhere [177]. In this section we are focusing on the α7nAChR, which KYNA directly inhibits as firstly reported by Hilmas et al. [178] and further confirmed by several in vitro and in vivo reports (for review see [179]). However, other studies also questioned this effect [180,181]. A recent review corroborated that KYNA can be considered a bona fide endogenous modulator for α7nAChR, but established as a complex phenomenon, depending on mostly methodological considerations [179].

The α7nAChR belongs to the neuronal type nAChRs and shares the five transmembrane subunit structure with this subfamily, but it consists of only α7 subunits, creating a homomeric structured ion channel [182]. Additionally, among the neural homomeric nAChRs, α7nAChR is the most abundant in the mammalian brain [182,183]. As all nAChRs, the α7nAChR is too a cation sensitive ion channel, with a high calcium permeability. They are expressed extensively in the cortex and hippocampus and in neurons of the mesostriatal dopaminergic system [182], which also overlaps with the expression of the cannabinoid receptors. Similar to the CB1 receptor [184], the α7nAChR are presynaptic receptors and modulate the release of GABA, dopamine, noradrenaline and serotonin neurotransmitters, however in contrast to CB1 it enhances their release [185]. Hence, the α7nAChR share a number of functions with the CB1R including learning, memory or nicotine addiction [182].

The α7nAChR and cannabinoids have been in focus in terms of cannabis abuse. Solinas et al. demonstrated that the blockade of α7nAChR reversed the discriminative effects of THC and the synthetic cannabinoid agonist WIN55,212-2. Additionally, the α7nAChR antagonist methyllycaconitine also reduced WIN55,212-2 self-administration and blocked THC-induced enhanced dopamine levels in the NAc shell, which is an important region of rewarding effects [186]. Importantly these effects were observed at doses that do not induce psychiatric side-effects or toxicity [186].

### 6.5. GPR35: A Possible Interactional Partner for Cannabinoid Receptors

Among the direct receptor targets of KYNA, the GPR35 stands out being the only metabotropic receptor. GPR35 was cloned as an “orphan” GPCR [32] and it is reported to couple to Gα_i/o_ and Gα13 type G-proteins [31,33,187,188,189]. KYNA has been the first endogenous agonist ligand to be described for GPR35 [31], also the presence of KYNA overlaps with the expression of GPR35 in many tissues and organs (e.g., spleen, colon, and brain) [190]. However, the GPR35 endogenous ligand designation of KYNA is still a matter of debate, mainly because it activates the receptor in very high micromolar concentrations and it has been reported to be significantly more potent on rat than human GPR35 [31]. Several other endogenous GPR35 ligands have been described [191], among them the lysophosphatidic acid is the other well studied endogenous ligand besides KYNA [192].

Both cannabinoid receptors and the GPR35 distribution overlaps in certain cells or tissues such as in leukocytes in the immune system (CB2 receptors), the gastrointestinal system (GI) or in neurons of DRG and both have similar signaling pathways (coupling to Gα_i/o_, inhibiting adenylate cyclase activity etc.). Although there are no data referring to GPR35 and CB1 receptor being co-localized or to functionally interact in these areas, there are studies indicating its possibility. For instance, in DRG neurons, there is evidence that both CB1 and GPR35 are co-localized or co-expressed together with the TRPV1 channel in the small DRG neurons [59,126]. Additionally, both GPR35 and CB1 receptors mediate peripheral nociception [191,193], which in case of GPR35 might be induced by KYNA [59]. Another potential region for an anatomical and functional interaction of GPR35 and cannabinoid receptors could be the small intestines, colon or stomach, where both receptors are expressed in significant quantities [31,66,127,194], however the cell specific expression of GPR35 within the GI system has not been established yet in contrast to cannabinoid receptors [66]. At the same time, both GPR35 and cannabinoid receptors have been implicated in inflammatory bowel disease [195,196]. Further, both GPR35 via KYNA and CB2 receptors expressed on leukocytes have been reported to be involved in leukocyte recruitment [197,198,199,200,201,202,203,204]. Finally, although KYNA is not implicated in this finding, a review suggested a linkage between GPR35 and cannabinoid receptors through the interconversion of their endogenous ligands, the 2-acyl lysophosphatidic acid (GPR35) and 2-AG (cannabinoid receptors) [191].

## 7. Final Remarks and Conclusions

In summary, both the endocannabinoid and kynurenine systems can interact with each other in several levels, which might be relevant to migraine. However, the data are so far limited and some areas of this interaction are yet undiscovered, for instance the cross-talk between the endocannabinoids and their enzymes with the kynurenine pathway. Nevertheless, previous successful preclinical or clinical studies in regard of other pathological disorders demonstrated that the endocannabinoid and kynurenine system are potential therapeutic targets. Presently, the well-described NMDA-CB1 receptor complex might be the most promising therapeutic target against migraine, by either manipulating the endogenous levels of KYNA and endocannabinoids (Figure 2) and/or using specific exogenous compounds to target this receptor complex.

It is important to further investigate the mechanisms and interactional partners involved in the cross-talk between the endocannabinoid and kynurenine system, as it will also possibly reveal more information regarding the individual function of these two systems and also their connections with migraine.

## Abbreviations

2-AG	2-arachidonoylglycerol
3-HA	3-hydroxyanthranilic acid
3-HK	3-hydroxy-l-kynurenine
AEA	anandamide
AMPA	α-amino-3-hydroxy-5-methyl-4-isoxazole propionic acid
ANA	anthranilic acid
cAMP	cyclic adenosine monophosphate
CB_1_	type 1 cannabinoid receptor
CB_2_	type 2 cannabinoid receptor
CFA	complete Freund’s Adjuvant
CGRP	calcitonin gene-related peptide
CSD	cortical spreading depression
DRG	dorsal root ganglion
FAAH	fatty acid amide hydrolase
GABA	γ-Aminobutyric acid
GPCR	G-protein coupled receptor
GPR35	G protein coupled receptor 35
HINT1	histidine triad nucleotide-binding protein 1
IDO	indoleamine 2,3-dioxygenase
KA1	*N*-(2-*N*,*N*-dimethylaminoethyl)-4-oxo-1*H*-quinoline-2-carboxamide hydrochloride
KA2	*N*-(2-*N*-pyrrolidinylethyl)-4-oxo-1*H*-quinoline-2-carboxamide hydrochloride
KATII	kynurenine aminotransferase II
KMO	kynurenine 3-monooxygenase
KP	kynurenine pathway
KYNA	kynurenic acid
KYNU	l-kynurenine hydrolase
L-KYN	l-kynurenine
MAGL	monoacylglycerol lipase
MAP	mitogen-activated protein kinases
MOR	μ opioid receptor
NAD^+^	nicotinamide adenine dinucleotid
NMDA	*N*-methyl-d-aspartate
NO	nitric oxide
NTG	nitroglycerin
NAc	nucleus accumbens
PAG	periaqueaductal grey
QUIN	quinolinic acid
TDO	tryptophan 2,3-dioxygenase
THC	Δ9-tetrahydrocannabinol
Trp	tryptophan
α7nAChR	α7 nicotinic acetylcholine receptor
σ1R	σ receptor type 1
